# Knowledge, Attitudes, and Practices Toward the Prevention of Hepatitis B Virus Infection Among Medical Students in Medina City, Kingdom of Saudi Arabia

**DOI:** 10.7759/cureus.48845

**Published:** 2023-11-15

**Authors:** Muayad S Albadrani, Abdullah M Abdulaal, Ahmed M Aljabri, Saleh S Aljohani, Salman F Aljohani, Mohammed A Sindi, Hassan K Jan, Hatim Alsaedi, Waleed M Alamri, Abdulrahman M Alharbi, Abdulaziz A Alraddadi

**Affiliations:** 1 Family and Community Medicine, College of Medicine, Taibah University, Medina, SAU; 2 College of Medicine, Taibah University, Medina, SAU; 3 College of Medicine, Alrayan Colleges, Medina, SAU

**Keywords:** medina, medical students, knowledge, prevention, infection, hepatitis b

## Abstract

Introduction: This study focuses on assessing the knowledge, attitudes, and practices related to Hepatitis B virus (HBV) prevention among medical students in Medina, Saudi Arabia. HBV is a significant global health concern, with a high prevalence in Saudi Arabia. Medical students due to their field, are at higher risk of exposure. Prior studies in Saudi Arabia show varied levels of awareness. This research aims to provide insights that can inform educational initiatives for this specific population.

Methods: This was a cross-sectional study conducted from June 2023 to September 2023 by using a pre-designed online questionnaire that was distributed among medical students in Medina. Data was analyzed using IBM Corp. Released 2020. IBM SPSS Statistics for Windows, Version 27.0. Armonk, NY: IBM Corp.

Results: This study included 307 participants. 67.8% of the participants correctly identified the link between HBV and liver cancer, and 77.5% recognized the transmission risk from carriers. 91.9% acknowledged the transmission via contaminated blood and fluids, and 88.9% recognized the risk from unsterilized instruments. Positive attitudes were observed, with 92.2% agreeing that following infection control guidelines would protect them at work. Practice scores were generally positive, including high rates of screening (57.3%) and adherence to infection control measures (90.2%). Knowledge scores correlated positively with attitude (rho = 0.204) and practice scores (rho = 0.390).

Conclusion: A significant proportion of participants had a strong understanding of HBV transmission and the importance of infection control measures. Positive attitudes towards infection control were prevalent, although some reluctance to provide care to HBV-infected individuals was noted.

## Introduction

Hepatitis B virus (HBV) is an enveloped, double-stranded (DNA) virus that is considered a global health problem [1,2]. Around the world, 290 million people are estimated to have chronic hepatitis B infection, which is the major cause of hepatitis mortality and morbidity [3]. According to the Saudi Ministry of Health (MOH), HBV infection ranks second among viral diseases after the chickenpox virus, with a prevalence of approximately 7%-8% [4,5]. HBV can pass from mother to fetus during birth, via parenteral means, and through sexual contact [1]. It can lead to acute hepatitis, chronic hepatitis, liver cirrhosis, and hepatocellular carcinoma (HCC) [2]. In 2015, the World Health Organization (WHO) set a high aim to eliminate hepatitis B infections by 90% and boost worldwide vaccination rates to 90% [6]. The risk of HBV infection via minor skin cuts and unintentional needle punctures is higher for students in the healthcare industry, especially those studying medicine [7]. A recent study conducted in 2019 among medical students in Al Qassim, Saudi Arabia, found that they had poor knowledge, attitudes, and practices regarding the risk of HBV infection [8]. However, another study conducted in Arar, Saudi Arabia, found medical students to have good knowledge of HBV infection [9]. Despite these data, there is limited information about popular perceptions of viral hepatitis, attitudes toward it, and practices, both globally and in the Kingdom of Saudi Arabia. Our study will be a good source of information about current knowledge regarding HBV, which can evaluate the need for educational programs in our targeted population.

## Materials and methods

Data were gathered through the use of a digital survey that contained inquiries intended to achieve the goals of the research project. A brief introduction outlining the objectives and importance of the study was included with the questionnaire. The questionnaire was created based on a predesigned questionnaire used for the same research purpose [9].a predesigned questionnaire used for the same research purpose [9].

To collect data, an electronic questionnaire was created using Google Forms. Data were gathered through questionnaire distribution, in which Medina medical students were sent a link to the study via social media. The link to a briefing on the project was supplied by the data collectors, who kindly asked the participants, who were medical students in Medina, to fill out the questionnaire.

The questionnaire is categorized into four different parts. The study participants' socio-demographic variables were covered in Section 1's questions. This part included questions about GPA, academic years, college, married status, and gender. In Section 2, the participants' knowledge of hepatitis viruses was assessed by a series of questions. Participants' attitudes toward hepatitis viruses were elicited by questions in Section 3. Finally, questions about participant practices for hepatitis virus self-defense were included in Section 4.

Statistical analysis: Both descriptive and inferential statistical analyses of the data were carried out. Simple descriptive statistics of the sociodemographic characteristics and other categorical variables in the form of frequencies and percentages were calculated and tabulated. For continuous variables, medians and IQRs (inter-quartile ranges) were reported as measures of central tendency and dispersion, respectively, owing to the relatively non-normal distribution of variables assessed by the Kolmogorov-Smirnov test (p<0.001).

Scoring was done for knowledge, attitude, and practices towards the prevention of hepatitis B virus infection. For the 11, 4, and 6 questions assessing the knowledge, attitude, and practices towards hepatitis B virus infection prevention, a score of 1 was assigned for each correct response, and these were summed up to calculate the total knowledge, attitude, and practice scores of each participant. Additionally, for the last question related to practices concerning vaccination status, a score of 1 was assigned to participants who had received one dose, 2 for two doses, and 3 for three doses. Thus, the total possible score of a participant ranged from 0-11, 0-4, and 0-8 for knowledge, attitude, and practices, respectively. The scores were compared among participants of different sociodemographic characteristics. The comparison involved inferential statistical analysis, namely the non-parametric Mann-Whitney U test and the Kruskal-Walli’s test. Furthermore, correlation was assessed between knowledge, attitude, and practice scores using the Spearman-rank correlation method. Significance was established at a p-value of 0.05, indicating a 95% confidence interval. All statistical calculations were performed using IBM Corp. Released 2020. IBM SPSS Statistics for Windows, Version 27.0. Armonk, NY: IBM Corp.

## Results

Sociodemographic characteristics

The sample consisted of 307 participants, with 41.0% males and 59.0% females. Regarding marital status, the majority were single (96.4%), while 2.6% were married, and 1.0% were divorced. In terms of academic affiliation, 66.1% were from Taibah University, and 33.9% were from Al-Rayan College of Medicine. Academic year distribution showed that the participants were spread across various stages of their medical education, with the highest representation from interns (23.1%), followed by third year (16.9%) and fifth-year (16.0%) students. The GPA distribution was diverse, with 48.9% having a GPA greater than 4.5, 38.4% in the 4.5 to 4.001 range, and smaller percentages in the other categories. These sociodemographic characteristics offer insight into the composition of the study population and provide a foundation for further analysis and interpretation of the data collected (Table 1).

**Table 1:** Sociodemographic Characteristics of the Participants

Variable		N	%
Gender	Male	126	41.0%
	Female	181	59.0%
Marital status	Single	296	96.4%
	Married	8	2.6%
	Divorced	3	1.0%
Group	Taibah University	203	66.1%
	Al-Rayan College of Medicine	104	33.9%
Academic Year	Third year	52	16.9%
	Sixth year	47	15.3%
	Second year	52	16.9%
	Intern	71	23.1%
	Fourth year	36	11.7%
	Fifth year	49	16.0%
GPA	4.5 to 4.001	118	38.4%
	3.51 to 4.00	32	10.4%
	3.5 to 2.5	7	2.3%
	>4.5	150	48.9%

Knowledge questions

Among the 307 participants, the majority demonstrated an accurate understanding of HBV infection. Specifically, 67.8% correctly identified that HBV can cause liver cancer, while 77.5% recognized that carriers of the virus can transmit the infection. Additionally, 91.9% of participants correctly acknowledged that HBV can be transmitted through contaminated blood and body fluids, and 88.9% recognized the risk associated with unsterilized syringes, needles, and surgical instruments. Furthermore, 76.2% correctly knew that a vaccine is effective in preventing HBV infection. Notably, 87.0% of participants were aware that HBV can be laboratory tested. The majority (81.4%) correctly knew that casual contact like handshaking cannot transmit the virus, and 61.2% knew that contact with an open wound or cut can spread the infection. Furthermore, while 76.2% were aware of the vaccine's preventive role, 76.2% were aware that HBV could be transmitted via unsafe sex (Table 2).

**Table 2:** Responses of the Participants to Knowledge Questions

Knowledge Question	Correct Answer	Responses of the Participants					
		Don't know		No		Yes	
		N	%	N	%	N	%
Hepatitis B Virus causes liver cancer	Yes	63	20.5%	36	11.7%	208	67.8%
Hepatitis B Virus carriers can transmit infection	Yes	41	13.4%	28	9.1%	238	77.5%
Hepatitis B Virus infection is spread by casual contact like hand shaking	No	33	10.7%	250	81.4%	24	7.8%
Hepatitis B Virus infection is spread by contact with an open wound / cut	Yes	63	20.5%	56	18.2%	188	61.2%
Hepatitis B Virus can be transmitted by contaminated blood and body fluids	Yes	16	5.2%	9	2.9%	282	91.9%
Virus be transmitted by unsterilized syringe, needle and surgical instruments	Yes	26	8.5%	8	2.6%	273	88.9%
Hepatitis B Virus can be transmitted by unsafe sex	Yes	38	12.4%	35	11.4%	234	76.2%
A vaccine can prevent Hepatitis B Virus infection	Yes	49	16.0%	24	7.8%	234	76.2%
Hepatitis B Virus can be laboratory tested	Yes	32	10.4%	8	2.6%	267	87.0%
Hepatitis B Virus has post-exposure prophylaxis	Yes	112	36.5%	18	5.9%	177	57.7%
Hepatitis B Virus can be cured/treated	Yes	94	30.6%	74	24.1%	139	45.3%

Attitude questions

The majority exhibited positive attitudes regarding infection control. Notably, 92.2% agreed that following infection control guidelines would protect them from HBV infection at work. However, 50.5% expressed discomfort with taking care of people with HBV, suggesting a lower level of acceptance and willingness to provide care. A minority (39.1%) agreed that all patients should be tested for HBV before receiving healthcare. However, a significant proportion (81.4%) disagreed that changing gloves during blood collection is a waste of time, highlighting the importance participants placed on proper infection control measures (Table 3).

**Table 3:** Responses of the Participants to Attitude Questions

Attitude Questions	Correct Attitude	Response of the Participants					
		Agree		Disagree		Don't know	
		N	%	N	%	N	%
Change of the gloves during blood collection is a waste of time	Disagree	40	13.0%	250	81.4%	17	5.5%
All patients should be tested for Hepatitis B Virus before they receive health care	Agree	120	39.1%	133	43.3%	54	17.6%
I Do not feel comfortable to take care of people with Hepatitis B Virus	Disagree	155	50.5%	113	36.8%	39	12.7%
Following infection control guidelines will protect me from being infected by Hepatitis B Virus at work	Agree	283	92.2%	5	1.6%	19	6.2%

Practice questions

Table 4 presents the practice-related responses of the study participants regarding hepatitis B virus (HBV) infection prevention. Notably, 57.3% of participants have been screened for HBV, and 90.2% always change gloves for each patient during blood collection. In terms of reporting needle-prick injuries, 70.4% always do so. Moreover, 62.2% have been vaccinated against HBV, with 33.9% receiving the recommended three doses. These results indicate generally positive practices, including a high willingness to undergo testing, adherence to infection control measures, and a significant vaccination rate.

**Table 4:** Responses of the Participants to Practice Questions

Practice Questions	Correct Practice	Responses of Participant	N	%
Have you ever screened for Hepatitis B Virus	Yes	No	131	42.7%
		Yes	176	57.3%
I always change gloves for each patient during blood taking	Yes	No	30	9.8%
		Yes	277	90.2%
Have you ever had a needle prick injury	No	No	253	82.4%
		Yes	54	17.6%
I always report for needle prick injury	Yes	No	91	29.6%
		Yes	216	70.4%
Have you been vaccinated against Hepatitis B Virus	Yes	No	116	37.8%
		Yes	191	62.2%
How many doses of Hepatitis B Virus vaccine did you receive	One/Two/Three Doses	Not received before	117	38.1%
		One dose	45	14.7%
		Three doses	104	33.9%
		Two doses	41	13.4%

Association of sociodemographic factors with knowledge, attitude and practice

Table 5 assesses the association between knowledge scores on Hepatitis B virus (HBV) and sociodemographic factors. The overall mean knowledge score was 8.11, with a median of 9.00 and an IQR of 7.00-10.00. Gender and marital status showed no significant differences in knowledge scores. Academic year, however, significantly influenced knowledge, with fifth-year students having the highest mean score (9.20) and second-year students the lowest (5.40) (p < 0.001). GPA did not significantly impact knowledge scores.

**Table 5:** Association of Knowledge Scores With Sociodemographic Characteristics ^K^independent Samples Kruskal-Wallis Test ^U^independent Samples Mann-Whitney U Test p<0.05, Significant*

Variable		Knowledge Score			
		Mean	Median	IQR	P value^K/U^
Overall		8.11	9.00	7.00-10.00	-
Gender	Female	8.20	9.00	7.00-10.00	0.670
	Male	7.98	9.00	7.00-10.00	
Marital status	Married	9.00	9.00	8.50-9.00	0.197
	Single	8.12	9.00	7.00-10.00	
	Divorced	4.33	2.00	2.00-9.00	
Group	Taibah University	8.33	9.00	8.00-10.00	0.104
	Al-Rayan College of Medicine	7.67	9.00	7.00-10.00	
Academic Year	Fifth year	9.20	9.00	8.00-10.00	<0.001*
	Sixth year	8.91	9.00	9.00-10.00	
	Fourth year	8.89	9.00	8.00-10.00	
	Intern	8.66	9.00	8.00-10.00	
	Third year	7.77	8.00	7.00-9.00	
	Second year	5.40	6.00	4.00-8.00	
GPA	3.5 to 2.5	8.43	10.00	7.00-10.00	0.824
	4.5 to 4.001	8.22	9.00	8.00-10.00	
	>4.5	8.05	9.00	7.00-10.00	
	3.51 to 4.00	7.91	9.00	7.00-9.00	

The overall mean attitude score was 2.50, with a median of 2.00 and an interquartile range (IQR) of 2.00-3.00. Gender, marital status, and group (university) did not show significant differences in attitude scores. The academic year also did not significantly impact attitude scores. However, GPA had a notable effect on attitudes, with students with a GPA greater than 4.5 displaying a higher mean attitude score of 2.67 compared to those with lower GPAs (p = 0.008) (Table 6).

**Table 6:** Association of Attitude Scores with Sociodemographic Characteristics ^K^independent Samples Kruskal-Wallis Test ^U^independent Samples Mann-Whitney U Test p<0.05, Significant*

Variable		Attitude Score			
		Mean	Median	IQR	P value^K/U^
Overall		2.50	2.00	2.00-3.00	-
Gender	Male	2.57	2.00	2.00-3.00	0.306
	Female	2.44	2.00	2.00-3.00	
Marital status	Married	2.88	3.00	2.50-3.00	0.393
	Single	2.49	2.00	2.00-3.00	
	Divorced	2.33	2.00	2.00-3.00	
Group	Al-Rayan College of Medicine	2.57	3.00	2.00-3.00	0.291
	Taibah University	2.46	2.00	2.00-3.00	
Academic Year	Sixth year	2.66	3.00	2.00-3.00	0.548
	Intern	2.56	2.00	2.00-3.00	
	Second year	2.54	2.00	2.00-4.00	
	Third year	2.44	2.00	2.00-3.00	
	Fifth year	2.41	2.00	2.00-3.00	
	Fourth year	2.28	2.00	2.00-3.00	
GPA	>4.5	2.67	3.00	2.00-4.00	0.008*
	3.51 – 4.00	2.50	2.00	2.00-3.00	
	4.5 – 4.001	2.31	2.00	2.00-3.00	
	3.5 - 2.5	2.00	2.00	1.00-3.00	

The overall mean practice score was 5.06, with a median of 5.00 and an IQR of 4.00-7.00. Gender and marital status did not significantly affect practice scores. However, the university of enrollment had a substantial impact, with Taibah University students showing higher practice scores (5.31) than Al-Rayan College students (4.56) (p < 0.001). Academic year also significantly influenced practice scores, with fourth-year students having the highest mean score (6.33) and second-year students the lowest (3.58) (p < 0.001). GPA had no significant effect on practice scores (Table 7).

**Table 7:** Association of Practice Scores With Sociodemographic Characteristics ^K^independent Samples Kruskal-Wallis Test ^U^independent Samples Mann-Whitney U Test p<0.05, Significant*

Variable		Practice Score			
		Mean	Median	IQR	P value^K/U^
Overall		5.06	5.00	4.00-7.00	-
Gender	Male	5.27	5.00	4.00-7.00	0.135
	Female	4.91	5.00	4.00-6.00	
Marital status	Married	5.63	6.00	4.00-7.50	0.721
	Single	5.04	5.00	4.00-7.00	
	Divorced	5.00	6.00	3.00-6.00	
Group	Taibah University	5.31	5.00	4.00-7.00	<0.001*
	Al-Rayan College of Medicine	4.56	4.00	3.00-5.50	
Academic Year	Fourth year	6.33	6.50	5.00-8.00	<0.001*
	Sixth year	6.00	6.00	5.00-7.00	
	Fifth year	5.78	6.00	4.00-7.00	
	Intern	5.41	5.00	4.00-7.00	
	Third year	3.63	4.00	3.00-4.50	
	Second year	3.58	3.00	3.00-5.00	
GPA	3.51 to 4.00	5.34	5.50	3.00-7.50	0.626
	3.5 to 2.5	5.29	5.00	5.00-7.00	
	4.5 to 4.001	5.10	5.00	4.00-6.00	
	>4.5	4.95	5.00	3.00-6.00	

Correlation of knowledge, attitude, and practice

The table shows that knowledge scores were positively correlated with both attitude scores (rho = 0.204, p < 0.01) and practice scores (rho = 0.390, p < 0.001). However, attitude and practice scores had a weaker positive but non-significant correlation (rho = 0.107, p = 0.061). These results indicate that individuals with higher knowledge scores tend to have more positive attitudes and engage in better practices related to HBV infection prevention (Table 8). 

**Table 8:** Correlation Between Knowledge, Attitude, and Practice Scores Correlation is significant at the 0.01 level (2-tailed).**

Correlations					
Variable			Knowledge Score	Attitude Score	Practice Score
Spearman's rho	Knowledge Score	Correlation Coefficient	1.000	.204**	.390**
		Sig. (2-tailed)	.	<0.001	<0.001
		N	307	307	307
	Attitude Score	Correlation Coefficient	.204**	1.000	.107
		Sig. (2-tailed)	<0.001	.	.061
		N	307	307	307
	Practice Score	Correlation Coefficient	.390**	.107	1.000
		Sig. (2-tailed)	<0.001	.061	.
		N	307	307	307

## Discussion

Our study involved 307 participants, predominantly single, from Taibah University and distributed across various academic years.

The findings on knowledge are quite promising. A considerable proportion of participants demonstrated a commendable understanding of HBV infection. They correctly identified critical aspects such as the potential for HBV to cause liver cancer, the diverse modes of transmission, the role of contaminated blood and body fluids, and the efficacy of vaccination. These results are indicative of the student’s strong foundation in HBV-related knowledge, which is vital for healthcare professionals who are at the forefront of patient care. Previous studies found similar levels of knowledge among medical students in a different region, underscoring the importance of HBV awareness in medical education [10,11].

Nonetheless, there were areas where knowledge gaps became evident. Although the majority understood that casual contact like handshaking does not transmit the virus, there was a knowledge deficit regarding potential transmission through contact with open wounds and the significance of practicing safe sexual behaviors [12]. These gaps highlight the need for targeted educational efforts to rectify these specific aspects of HBV transmission. Tailored training programs could further enhance the students’ grasp of these critical concepts.

The study revealed predominantly positive attitudes among participants regarding infection control. Most agreed that adhering to infection control guidelines would protect them from HBV infection while working. This positive attitude demonstrates an awareness of the importance of adhering to established protocols for infection prevention within healthcare settings [8].

However, a notable portion expressed discomfort regarding providing care to individuals with HBV. This raises concerns about their willingness to care for HBV patients, potentially leading to a suboptimal patient-care experience. Addressing this aspect is essential, as medical professionals should be equipped with the knowledge and emotional readiness to treat all patients without discrimination. The discomfort expressed by participants in caring for HBV patients may differ from the results of a study by Ali et al. (2017), which showed more positive attitudes [13].

Moreover, a significant percentage did not agree with the idea of testing all patients for HBV before healthcare, indicating the need for further education on the importance of early detection and prevention [14]. This finding underscores the significance of developing comprehensive educational interventions that highlight the benefits of testing in a healthcare context.

On a positive note, the majority disagreed with the notion that changing gloves during blood collection is a waste of time. This suggests that participants place great importance on proper infection control measures. This positive attitude is a key asset in maintaining a safe healthcare environment [9].

The practices related to HBV infection prevention among the participants were generally positive. A substantial proportion had been screened for HBV, consistently changed gloves during patient care, reported needle prick injuries, and had been vaccinated against HBV. These practices reflect their commitment to maintaining infection control measures and their personal safety. Of particular significance is the high vaccination rate, which not only safeguards healthcare workers but also prevents them from becoming carriers and potentially transmitting the virus to patients [15].

Medical institutions should focus on reinforcing the existing knowledge of students while addressing specific gaps in understanding. Targeted educational programs and workshops should be designed to improve knowledge, particularly in areas where gaps were identified. Providing students with comprehensive information about HBV transmission modes, including the risk associated with open wounds and the importance of safe sexual practices, is essential [16].

Interventions are also needed to improve attitudes, with a focus on reducing discomfort in caring for HBV patients and emphasizing the importance of patient testing. Programs that promote empathy and reduce stigma in healthcare settings can be particularly valuable [17]. Additionally, emphasizing the importance of early testing and diagnosis for HBV should be part of the educational efforts.

Moreover, the positive practices observed suggest a solid foundation for creating a safer healthcare environment, but these practices should be continually reinforced through training and regular reminders. The high vaccination rate is a positive aspect, and efforts should be made to ensure that all healthcare students and workers are fully vaccinated against HBV [18].

The correlation analysis revealed that higher knowledge scores were positively correlated with more positive attitudes and better practices. This suggests that improving knowledge may have a cascading effect, positively influencing attitudes and practices. It reinforces the importance of knowledge as a foundational element in improving overall attitudes and practices. These findings can guide future interventions and educational strategies aimed at enhancing HBV prevention among medical students [19,20].

Several limitations must be acknowledged in this study. The study focused exclusively on medical students in Medina, Saudi Arabia, and, therefore, the findings may not be generalizable to healthcare students in different regions or to other healthcare professionals. Additionally, self-reported data was used for knowledge, attitudes, and practices, which may introduce the potential for social desirability bias. The study also did not explore in-depth qualitative insights into the reasons behind certain attitudes or practices, which could provide a more comprehensive understanding. Furthermore, the cross-sectional nature of the study limits the ability to establish causality or assess changes over time.

## Conclusions

The study reveals a commendable level of knowledge about HBV and a generally positive attitude towards infection control measures among the participants. Their practices also demonstrated a high level of adherence to recommended guidelines, including a significant rate of HBV vaccination. These positive outcomes are promising for the future of healthcare in Medina. However, certain gaps in knowledge, particularly related to specific modes of HBV transmission and expressed discomfort in caring for HBV patients, call for targeted educational interventions. Efforts should be made to reinforce knowledge and further enhance attitudes to ensure healthcare students are fully equipped to provide comprehensive and non-discriminatory care to HBV patients.
